# Conditional Covariances for the Signal Lag Measurements in Fluoroscopic Imaging

**DOI:** 10.3390/diagnostics12010087

**Published:** 2021-12-31

**Authors:** Eunae Lee, Dong Sik Kim

**Affiliations:** Department of Electronics Engineering, Hankuk University of Foreign Studies, Seoul 17035, Korea; eunae.lee92@hufs.ac.kr

**Keywords:** conditional covariance, fluoroscopic imaging, lag correction factor, nonuniform temporal gain, upper-lower correction

## Abstract

In fluoroscopic imaging, we can acquire X-ray image sequences using a flat-panel dynamic detector. However, lag signals from previous frames are added to the subsequently acquired images and produce lag artifacts. The lag signals also inflate the measured noise power spectrum (NPS) of a detector. In order to correct the measured NPS, the lag correction factor (LCF) is generally used. However, the nonuniform temporal gain (NTG), which is from inconsistent X-ray sources and readout circuits, can significantly distort the LCF measurements. In this paper, we propose a simple scheme to alleviate the NTG problem in order to accurately and efficiently measure the detector LCF. We first theoretically analyze the effects of NTG, especially on the correlation-based LCF measurement methods, where calculating the correlation coefficients are required. In order to remove the biases due to NTG, a notion of conditional covariance is considered for unbiased estimates of the correlation coefficients. Experiments using practical X-ray images acquired from a dynamic detector were conducted. The proposed approach could yield accurate LCF values similarly to the current approaches of the direct and U-L corrections with a low computational complexity. By calculating the correlation coefficients based on conditional covariance, we could obtain accurate LCF values even under the NTG environment. This approach does not require any preprocessing scheme of the direct or U-L correction and can provide further accurate LCF values than the method of IEC62220-1-3 does.

## 1. Introduction

In fluoroscopic imaging, flat-panel (FP) dynamic detectors can acquire X-ray image sequences with frame rates higher than 300 frames per second (fps) [1]. However, the sequentially acquired images have lag signals from previous frames [1,2]. For indirect detectors, trapping charges in the amorphous structure of the thin-film-transistor (TFT) panel and incomplete reads are the main causes of lag signals [3,4,5,6]. The lag artifacts appear in the form of temporal blurring and ghosting in fluoroscopic imaging [7,8,9,10,11,12,13].

In measuring the noise power spectrum (NPS) for a dynamic detector [14,15,16], the lag signal lowers the NPS curve. Conventional approaches for correcting the measured NPS are based on using the lag correction factor (LCF) [13,17,18,19,20,21]. Busse et al. [19,20] used a temporal power spectral density (PSD) method to measure LCF using steady-state images based on a moving average model of order *L* (MA(*L*)) for the lag signals. This PSD method is adopted in the IEC62220-1-3 standard [22]. Recently, Kim and Lee [21] proposed an LCF measurement method using a series of the Pearson correlation coefficients based on the MA(*L*) model. Based on an autoregressive model of order 1 (AR(1)), Matsunaga et al. [17] considered LCF measurements. Granfors and Aufrichtig [18], as well as Kim and Lee [13], considered the means of transient decaying images after the X-ray turns off.

Because of the nonuniform temporal gain (NTG) from inconsistent X-ray sources and readout circuits, accurately measuring PSD curves as well as correlations for LCF is difficult. In particular, the PSD method [19] is very sensitive to NTG because the temporal spectrum at low frequencies has unusually high values. Hence, applying a gain correction is important in obtaining an accurate LCF [19]. Compared to the PSD case, the correlation method [21] is less sensitive to NTG even though a gain correction is required. On the other hand, the mean-based methods are generally insensitive to NTG [13,18].

In this paper, we analyze effects of NTG on the correlation-based methods [21]. Formulating a mathematical NTG model, the effects on measuring the correlations are observed. We also theoretically observe that a scheme called the upper-lower (U-L) correction can efficiently alleviate the NTG problem. We then propose an LCF measurement method based on the correlation method considering estimates from conditional covariances. This algorithm can efficiently measure LCF without applying any gain correction schemes. We extensively conducted experiments using real X-ray images acquired from a dynamic detector.

This paper is organized in the following way. In Section 2, the definitions on LCF for the MA(*L*) and AR(1) models are introduced. An NTG model is formulated and theoretical analyses are conducted in Section 3, and an LCF measurment algorithm is proposed in Section 4. Experimental results are shown in Section 5, and the paper is concluded in the last section.

## 2. Lag Correction Factors

In this section, we formulate two lag models based on MA(*L*) and AR(1) for introducing the LCF definitions, respectively.

For a pixel position u, let $g_{n}\left[u\right]$ denote a signal that is independent and identically distributed. We first consider a linear lag model of MA(*L*). The MA(*L*) model [18] with a signal $f_{n}$ is defined as

(1) $$f_{n}\left[u\right]=\sum_{l=0}^{L}g_{n-\ell }\left[u\right]h_{\ell },\;for\;u\in {\left\{0,\ldots ,U-1\right\}}^{2},$$

where $h_{\ell }$ is a causal system, such that $\sum_{\ell =0}^{L}h_{\ell }=1$ with nonnegative $h_{\ell }$. Here, $f_{n}$ has $U^{2}$ pixels and represents the *n*th X-ray image acquired under specified irradiation conditions with uniform intensity. Let $I_{f}$ and $I_{g}$ denote the periodogram means of $f_{n}$ and $g_{n}$, respectively [23]. The periodogram means then satisfy $I_{f}=r_{MA}I_{g}$, where $r_{MA}$ is the LCF under the MA(*L*) model and is defined as $r_{MA}:=\sum_{\ell =0}^{L}h_{\ell }^{2}$. By using this LCF, we can asymptotically correct the measured NPS to obtain the true NPS from $NPS_{g}\approx NPS_{f}/r_{MA}$ [24], where $NPS_{g}$ and $NPS_{f}$ are the NPS curves of *g* and *f*, respectively. Let $\rho_{\ell }$ denote the autocorrelation of $f_{n}$ with the frame lag *ℓ* defined as

(2) $$\begin{array}{c} \rho_{\ell }:=\frac{\mathrm{Cov}\{f_{0},f_{\ell }\}}{\mathrm{Var}\{f_{0}\}},\;for\;\ell =0,\ldots ,L. \end{array}$$

Kim and Lee [21] showed that the LCF of $r_{MA}$ can be a function of $\rho_{\ell }$ as

(3) $$\begin{array}{c} r_{MA}=\frac{1}{2\sum_{\ell =0}^{L}\rho_{\ell }-1}. \end{array}$$

Obtaining the autocovariance of $f_{n}$ is enough to calculate the autocorrelation $\rho_{\ell }$ of Equation (2) and thus the LCF of Equation (3).

We now consider a linear lag model based on AR(1). The acquired image $f_{n}$ can be described from the AR(1) model point of view [17] as

(4) $$f_{n}\left[u\right]=af_{n-1}\left[u\right]+\left(1-a\right)g_{n}\left[u\right],for\;u\in {\left\{0,\ldots ,U-1\right\}}^{2}.$$

From Equation (4), we can obtain a relationship of $I_{f}\left[v\right]=r_{AR}I_{g}\left[v\right]$, where $r_{AR}$ is the LCF under the AR(1) model and is defined as $r_{AR}:=\left(1-a\right)/\left(1+a\right)$. We can obtain *a* of AR(1) from the autocorrelation as $a=\rho_{1}$, and the resultant $r_{AR}$ can also be a function of $\rho_{1}$ as

(5) $$r_{AR}=\frac{1-\rho_{1}}{1+\rho_{1}}.$$

Note that we can also obtain *a* from image means for transient decaying frames after the X-ray turns off. By using the LCF of Equation (5), we can also correct the measured NPS to obtain the true NPS. For relatively small values of $\rho_{1}$, $\left(1-\rho_{1}\right)/\left(1+\rho_{1}\right)\approx 1/\left(2\rho_{1}+1\right)$ holds. In other words, we can obtain an approximation of $r_{MA}\approx r_{AR}$ for L=1 from Equations (3) and (5) if $\rho_{1}$ is relatively small and thus obtain an approximate LCF value using a coefficient $\rho_{1}$, as discussed by Granfors and Aufrichtig [18].

## 3. Nonuniform Temporal Gain Model

For appropriately measured autocorrelations, measuring LCF from Equations (3) or (5) can be insensitive to various noises compared to the PSD method [19]. In this section, theoretical analyses are conducted on this sensitivity property to NTG.

In calculating $\rho_{\ell }$, various noises, such as NTG, can make accurate LCF measurements difficult. In order to mathematically describe NTG, we introduce a weakly stationary random sequence $\gamma_{n}$ with mean $E\{\gamma_{n}\}=1$ and variance $\mathrm{Var}\{\gamma_{n}\}$, and we modify the image model of Equations (1) or (4) considering NTG as [21]

(6) $$\begin{array}{c} q_{n}\left[u\right]:=\gamma_{n}f_{n}\left[u\right],\;for\;u\in {\left\{0,\ldots ,U-1\right\}}^{2}, \end{array}$$

where $q_{n}$ is a signal with NTG, and $\gamma_{n}$ is independent of the pixel values $f_{n}$. In the NTG model of Equation (6), we can practically assume that $1\gg \mathrm{Var}\{\gamma_{n}\}$. Hence, we can obtain an approximation of $E\left\{\gamma_{n}^{2}\right\}=1+\mathrm{Var}\left\{\gamma_{n}\right\}\approx 1$. The signal-to-noise ratio, $E\left\{\gamma_{n}\right\}/\sqrt{\mathrm{Var}\{\gamma_{n}\}}$, is very high in practical fluoroscopic imaging. Hence, this NTG can be ignored in the mean-based LCF measurement methods [13,17,18]. However, the PSD or correlation-based methods can be very sensitive to NTG even though its strength is quite weak [21].

In estimating the autocovariance $\mathrm{Cov}\{f_{0},f_{\ell }\}$ for $\rho_{\ell }$ in Equation (2), we can instead measure $\mathrm{Cov}\{q_{0},q_{\ell }\}$ using the acquired image $q_{n}$ and can have a bias of $\mu^{2}\mathrm{Cov}\left\{\gamma_{0},\gamma_{\ell }\right\}$ as derived in Appendix A. Hence, we cannot accurately calculate $\rho_{n}$ for the LCF values of Equations (3) and (5) if we use the autocovariance of $q_{n}$ due to the bias from NTG.

To reduce the biases from NTG, we can estimate the gain $\gamma_{n}$ from a conditional mean $\gamma_{n}=E\left\{q_{n}|\gamma_{n}\right\}/\mu$ and can then directly correct NTG from dividing $q_{n}$ by $\gamma_{n}$ [19]. Instead of this direct correction, we can use a notion from the image differences, as described by Kim [25] and Kim and Lee [26], for a weak gain. For an image $q_{n}$ with $U^{2}$ pixels, the image difference $\Delta q_{n}$ between the upper and lower pixels can be calculated as

(7) $$\begin{array}{c} \Delta q_{n}\left[u\right]:=\frac{1}{\sqrt{2}}\left[q_{n}\left[u\right]-q_{n}\left[\bar{u}\right]\right], \end{array}$$

for the upper image positions of u∈{0,⋯,U−1}×{0,⋯,U/2−1}, where $\bar{u}:=u+\left(0,U/2\right)$ implies the lower image part. In Equation (7), we assume that the pixels that are separated by U/2 pixels are mutually uncorrelated or are α-mixing [27]. The autocovariance of $\Delta q_{n}$ approximately satisfies $\mathrm{Cov}\left\{\Delta q_{0},\Delta q_{\ell }\right\}\approx \mathrm{Cov}\left\{f_{0},f_{\ell }\right\}$, as shown in Appendix B. Hence, by conducting a preprocessing on the difference $\Delta q_{n}$, we can accurately estimate LCF regardless of the influence of NTG. Here, we call the preprocessing the U-L correction. The measured autocorrelation can now be given as $\rho_{\ell }\approx \mathrm{Cov}\left\{\Delta q_{0},\Delta q_{\ell }\right\}/\mathrm{Var}\left\{\Delta q_{0}\right\}$ using the difference $\Delta q_{n}$ of Equation (7).

## 4. Estimates Based on Conditional Covariance

In this section, we propose a method based on conditional covariance for an unbiased estimate of $\rho_{\ell }$ in Equation (2) to alleviate the NTG problem besides the direct and U-L corrections. The conventional schemes of the direct and U-L corrections are applied to the input signal as preprocessing approaches. Estimates of $\mathrm{Cov}\{f_{0},f_{\ell }\}$ are next obtained to calculate $\rho_{\ell }$. However, the proposed method can obtain unbiased estimates of $\mathrm{Cov}\{f_{0},f_{\ell }\}$ even for the NTG environment based on conditional covariance.

A conditional covariance $E\{\mathrm{Cov}\left\{q_{0},q_{\ell }|\gamma_{0},\gamma_{\ell }\right\}\}$ can be expanded as

(8) $$\begin{array}{c} E\left\{\mathrm{Cov}\{q_{0},q_{\ell }|\gamma_{0},\gamma_{\ell }\}\right\}=\left(E^{2}\left\{\gamma_{0}\right\}+\mathrm{Cov}\left\{\gamma_{0},\gamma_{\ell }\right\}\right)\mathrm{Cov}\left\{f_{0},f_{\ell }\right\} \end{array}$$

for ℓ=0,…,L. From the assumption on $\gamma_{n}$, $E^{2}\left\{\gamma_{0}\right\}+\mathrm{Cov}\left\{\gamma_{0},\gamma_{\ell }\right\}\approx E^{2}\left\{\gamma_{0}\right\}=1$ holds. Hence, we can obtain an approximation for the conditional covariance as $E\left\{\mathrm{Cov}\left\{q_{0},q_{\ell }|\gamma_{0},\gamma_{\ell }\right\}\right\}\approx \mathrm{Cov}\left\{f_{0},f_{\ell }\right\}$ and use the conditional covariance as an unbiased estimate of $\mathrm{Cov}\{f_{0},f_{\ell }\}$ for calculating $\rho_{\ell }$. We now introduce the proposed algorithm as follows. For *N* frames of $q_{n}$, where N>L, we can asymptotically estimate the covariances on $q_{n}$ from empirical sum of $q_{n}\left[u\right]$ as *N* and *U* increase. Using the *N* image samples of $q_{n}\left[u\right]$, an empirical covariance,

(9) $$\begin{array}{c} \frac{1}{U^{2}\left(N-\ell \right)-1}\sum_{n=0}^{N-\ell -1}\sum_{u}\left[q_{n}\left[u\right]-\mu \left(\ell \right)\right]\left[q_{n+\ell }\left[u\right]-\mu \left(\ell \right)\right], \end{array}$$

where μ is an empirical mean defined as $\mu \left(\ell \right):=U^{-2}{\left(N-\ell \right)}^{-1}\sum_{n=0}^{N-\ell -1}\sum_{u}q_{n}\left[u\right]$, can be used as an estimate of $\mathrm{Cov}\{q_{0},q_{\ell }\}$. As mentioned in Appendix A, the estimate from Equation (9) has a bias of $\mu^{2}\mathrm{Cov}\left\{\gamma_{0},\gamma_{\ell }\right\}$ in estimating $\mathrm{Cov}\{f_{0},f_{\ell }\}$.

On the other hand, the conditional covariance of Equation (8) can be empirically estimated from an empirical estimate:

(10) $$\begin{array}{c} \frac{1}{N-\ell }\sum_{n=0}^{N-\ell -1}\left[\frac{1}{U^{2}-1}\sum_{u}\left[q_{n}\left[u\right]-\nu \left(n\right)\right]\left[q_{n+\ell }\left[u\right]-\nu \left(n\right)\right]\right], \end{array}$$

where ν is an empirical mean defined as $\nu \left(n\right):=U^{-2}\sum_{u}q_{n}\left[u\right]$. Based on the conditional covariance of (8) and its empirical estimate Equation (10), we can calculate the autocorrelation $\rho_{\ell }$ of Equation (2) and obtain the LCF values from Equations (3) or (5). The proposed LCF measurement method within a framework of the correlation-based methods [17,21] is now summarized as follows.

Conditional Covariance Algorithm:

(1) For a given X-ray image sequence $q_{n}\left[u\right]$, calculate the empirical estimate with Equation (10) for the conditional covariance $E\{\mathrm{Cov}\left\{q_{0},q_{\ell }|\gamma_{0},\gamma_{\ell }\right\}\}$.
(2) Using the empirical estimates, calculate $\rho_{\ell }$ to obtain the LCF of $r_{MA}$ from (3) or $r_{AR}$ from Equation (5).

From this Conditional Covariance Algorithm with the correlation-based methods, we can obtain accurate LCF values without applying the direct or U-L correction preprocessing.

## 5. Experimental Results

In this section, we introduce experimental results for analyzing the NTG effect on estimating LCF. We used a CsI(Tl)-scintillator FP dynamic detector (DRTECH Co., Ltd., Sungnam-si, Korea), where amorphous In-Ga-Zn-O thin-film transistors control the photodiode pixels. At frame rates of 10 fps and 30 fps, X-ray image sequences for various incident exposures were acquired under the RQA 5 condition of IEC62220-1-3 [22] and a continuous fluoroscopy mode for a continuous X-ray source.

**Figure 1:**
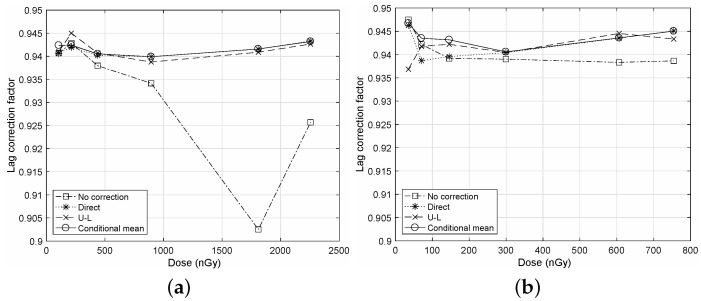
Comparisons of the LCF measurements for the different NTG corrections. The LCF values are calculated from $r_{MA}$ of Equation (3) derived in the correlation method [21] for MA(*L*). A sequence has N=128 images and L=N−1. (**a**) Frame rate of 10fps; (**b**) Frame rate of 30fps.

In Figure 1, we illustrate a comparison of the LCF measurements for the different NTG corrections to show the correction performance of the proposed algorithm. Here, the calculated autocorrelation $\rho_{\ell }$ are applied to $r_{MA}$ of Equation (3) to obtain LCF. We can observe that the proposed algorithm yields LCF values that are very close to that of the direct correction as “Conditional mean” and “Direct”. However, without the NTG correction, we can observe from “No correction” that the resultant LCF values have significant deviations from those of the corrected cases due to the bias term described in Appendix A. For both cases of frame rates in Figure 1a,b, we can observe similar results on the proposed algorithm.

## 6. Discussions

**Figure 2:**
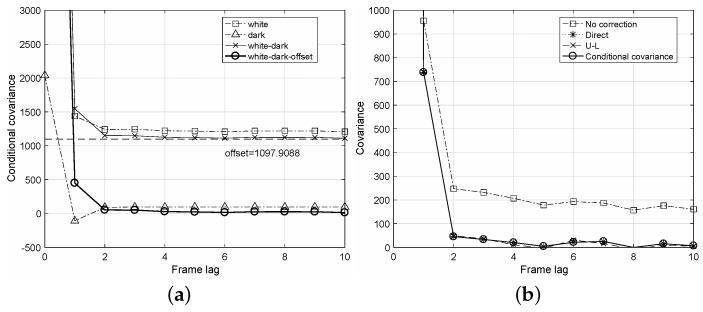
Estimated conditional covariances with respect to the frame lag *ℓ*. The frame rate was 10 fps, and the dose was 1809.5 nGy. (**a**) Conditional covariances of the white and dark images in the proposed conditional covariance algorithm; (**b**) Comparison of the NTG correction schemes and the proposed conditional covariance algorithm.

**Figure 3:**
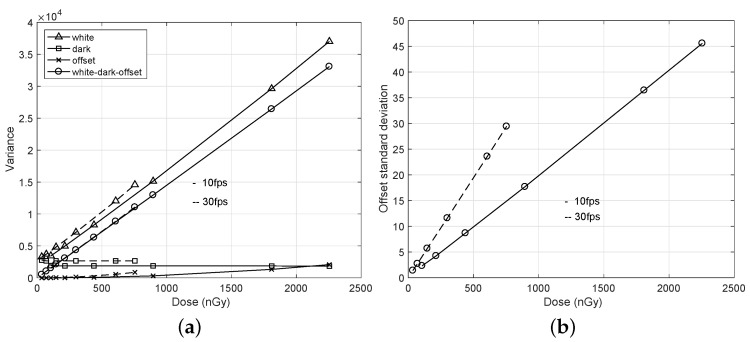
Observation of the photon noise and the exposure leak for a dynamic detector in Figure 1. Both the variance electric noise and variance offset are extracted from the variances of the white images to observe a linearity of the photon noise with respect to the incident dose. (**a**) Variance curves; (**b**) Standard deviation curves of the variance offset.

In practical calculations of the conditional covariance $E\{\mathrm{Cov}\left\{q_{0},q_{\ell }|\gamma_{0},\gamma_{\ell }\right\}\}$, further careful steps are required. Let the white image imply an image acquired at uniform exposure to the detector and the dark image imply an image acquired without exposures. From Step 1) of the algorithm, we first calculate the conditional covariances for both white and dark sequences, respectively, as “white” and “dark” in Figure 2a. Here, considering the dark conditional covariance is important because it can have negative values. We next calculate their difference as “white-dark” and then obtain a variance offset of the difference for relatively large frame lags of *ℓ*. The final conditional covariance is obtained by subtracting the variance offset from the difference as “white-dark-offset” in Figure 2a. Note that the variance offset in the conditional covariance occurs because of a fixed pattern noise, which is independent of the image frames [21]. As shown in Figure 3a, extracting the variance offset can guarantee a linearity of the variance curve as “white-dark-off”. The standard deviation of this noise, i.e., the root square of the variance offset, is proportional to the incident dose, as shown in Figure 3b. In Figure 2b, estimated conditional covariances are compared. Without any NTG corrections, the conditional covariance curve shows a bias due to NTG. The proposed algorithm could achieve a curve that is very close to those of the conventional direct and U-L corrections.

**Figure 4:**
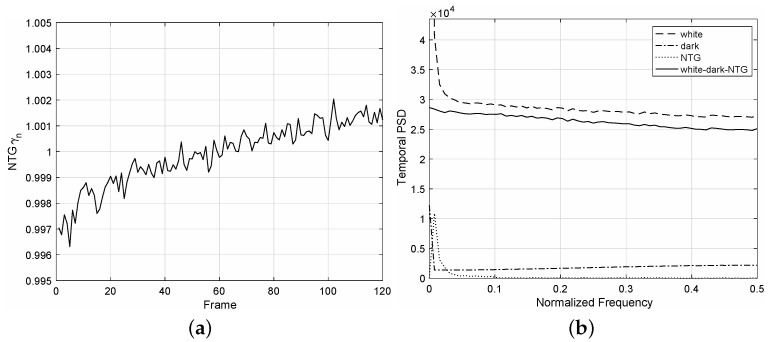
Example of NTG $\gamma_{n}$ (the frame rate is 10 fps, and the dose is 1809 nGy). (**a**) NTG curve of $\gamma_{n}$ with respect to the frame; (**b**) Temporal PSD curve of NTG.

For an image sequence acquired at an incident dose of 1809.5 nGy, we estimated the NTG signal $\gamma_{n}$ from the conditional mean $\gamma_{n}=E\left\{q_{n}|\gamma_{n}\right\}/\mu$. As shown in Figure 4a, the NTG curve $\gamma_{n}$ is noisy and is even slightly increasing. Hence, the temporal PSD of $\gamma_{n}$ has high values, especially at low frequencies, as shown in Figure 4b, and these values can produce high errors in obtaining LCF from the PSD method [19,21]. The variance of $\gamma_{n}$ satisfies $\mathrm{Var}\left\{\gamma_{n}\right\}\approx 1.509\times 10^{-6}\ll 1$, and thus, the assumption $E\left\{\gamma_{n}^{2}\right\}=1+\mathrm{Var}\left\{\gamma_{n}\right\}\approx 1$ is practically reasonable. The bias in estimating $\mathrm{Var}\{f_{n}\}$ from $\mathrm{Var}\{q_{n}\}$ is $\mu^{2}\mathrm{Var}\left\{\gamma_{n}\right\}\approx 344.6$ for a signal mean of μ = 15,110. From the proposed algorithm, the LCF is 0.9416, which is similar to those of the direct and U-L corrections. If we do not apply any NTG correction schemes, then the LCF is given as 0.9025, which is significantly lower than the proposed case. As a comparison, the line-estimate method [13], which is based on means, yields 0.9398.

**Figure 5:**
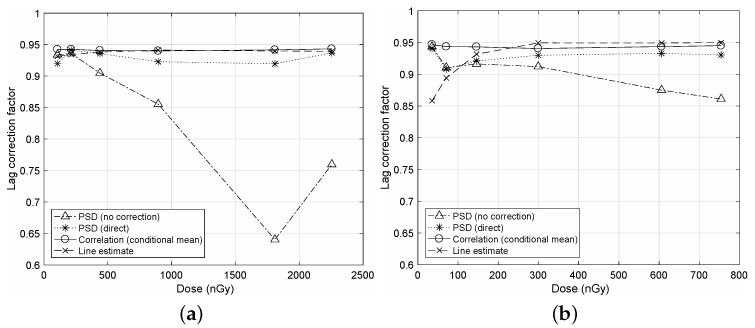
Comparison of the LCF measurements. “PSD (no correction)” is the method of IEC62220-1-3 without the NTG correction, and “PSD (direct)” is the PSD method with the direct correction [22]. “Correlation (conditional mean)” is the proposed algorithm, and “Line estimate” is the line estimate method [13]. (**a**) Frame rate of 10 fps; (**b**) Frame rate of 30 fps.

In Figure 5, a comparison on the NTG correction for the PSD method, which is adopted in IEC62220-1-3, is illustrated. As previously mentioned in the temporal PSD of Figure 4b, we notice that the PSD method is very sensitive to NTG as “PSD (no correction)”. Hence, the direct or U-L correction is required for the PSD method as “PSD (direct)”. Even though the NTG correction is conducted, the PSD method showed deviations from the proposed case. The LCF values from the proposed algorithm are very close to those of the line estimate method [13]. Note that the line estimate method is insensitive to NTG but uses transient decaying image frames after the X-ray tube tunes off from a continuous X-ray source.

## 7. Conclusions

In this paper, the NTG effect on the correlation-based LCF measurement methods was observed both theoretically and experimentally. A theoretical observation on the U-L correction to alleviate the NTG problem was also performed. We next proposed an unbiased estimate of autocorrelations based on the conditional covariance for the correlation-based LCF measurement method under the NTG environment. The proposed algorithm can be implemented without any preprocessing steps of the conventional NTG corrections and can obtain LCF more efficiently and accurately by replacing the method of IEC62220-1-3.

## Data Availability

Not applicable.

## Abbreviations

The following abbreviations are used in this manuscript:

AR	Autoregressive
FP	Flat panel
LCF	Lag correction factor
MA	Moving average
NPS	Noise power Spectrum
NTG	Nonuniform temporal gain
PSD	Power spectral density
U-L	Upper-lower

## Appendix A. Autocovariance of *q_n_*

From the law of total covariance, the autocovariance of $q_{n}$ can be expanded as

(A1) $$\begin{array}{c} \mathrm{Cov}\left\{q_{0},q_{\ell }\right\}=E\left\{\mathrm{Cov}\{q_{1},q_{\ell }|\gamma_{0},\gamma_{\ell }\}\right\}+\mathrm{Cov}\left\{E\left\{q_{0}|\gamma_{0},\gamma_{\ell }\right\},E\left\{q_{\ell }|\gamma_{0},\gamma_{\ell }\right\}\right\}, \end{array}$$

for ℓ=0,…,L, and can be rewritten as

(A2) $$\begin{array}{c} \mathrm{Cov}\left\{q_{0},q_{\ell }\right\}=\left(E^{2}\left\{\gamma_{0}\right\}+\mathrm{Cov}\left\{\gamma_{0},\gamma_{\ell }\right\}\right)\mathrm{Cov}\left\{f_{0},f_{\ell }\right\}+E^{2}\left\{f_{0}\right\}\mathrm{Cov}\left\{\gamma_{0},\gamma_{\ell }\right\}. \end{array}$$

From the assumption $E^{2}\left\{\gamma_{0}\right\}\gg \mathrm{Var}\left\{\gamma_{0}\right\}$, where $E\{\gamma_{0}\}=1$, we can obtain an approximation:

(A3) $$\begin{array}{c} \mathrm{Cov}\left\{q_{0},q_{\ell }\right\}\approx \mathrm{Cov}\left\{f_{0},f_{\ell }\right\}+\mu^{2}\mathrm{Cov}\left\{\gamma_{0},\gamma_{\ell }\right\}. \end{array}$$

Note that the bias $\mu^{2}\mathrm{Cov}\left\{\gamma_{0},\gamma_{\ell }\right\}$ can be removed for signals with a mean of zero.

## Appendix B. Autocovariance of Δ*q_n_*

From $E\{\Delta q_{n}\}=0$, the autocovariance of $\Delta q_{n}$ satisfies the following relationship:

(A4) $$\begin{array}{cc} \mathrm{Cov}\{\Delta q_{0}\left[u\right],\Delta q_{\ell }\left[u\right]\} & \;=\frac{1}{2}E\left\{\gamma_{0}\gamma_{\ell }\right\}E\left\{\left(f_{0}\left[u\right]-f_{0}\left[\bar{u}\right]\right)\left(f_{\ell }\left[u\right]-f_{\ell }\left[\bar{u}\right]\right)\right\}\;\\ \; & \;=\left(E^{2}\left\{\gamma_{0}\right\}+\mathrm{Cov}\left\{\gamma_{0},\gamma_{\ell }\right\}\right)\mathrm{Cov}\left\{f_{0},f_{\ell }\right\}, \end{array}$$

for ℓ=0,…,L. Hence, we can have an approximation of $\mathrm{Cov}\left\{\Delta q_{0},\Delta q_{\ell }\right\}\approx \mathrm{Cov}\left\{f_{0},f_{\ell }\right\}$. Note that there is no bias in estimating the autocovariance of $f_{n}$ from $\Delta q_{n}$.
